# FAP-α (Fibroblast activation protein-α) is involved in the control of human breast cancer cell line growth and motility via the FAK pathway

**DOI:** 10.1186/1471-2121-15-16

**Published:** 2014-05-21

**Authors:** Jun Jia, Tracey Amanda Martin, Lin Ye, Wen Guo Jiang

**Affiliations:** 1 Cardiff University-Peking University Cancer Institute, Cardiff University School of Medicine, Cardiff CF14 4XNWales, UK; 2 The Breast Oncology Department, Beijing Cancer Hospital, Peking University School of Oncology, Beijing 100142, China

**Keywords:** FAP-α, FAK, Breast Cancer, Growth, Migration

## Abstract

**Background:**

Fibroblast Activation Protein alpha (FAP-α) or seprase is an integral membrane serine peptidase. Previous work has not satisfactorily explained both the suppression and promotion effects that have been observed in cancer. The purpose of this work was to investigate the role of FAP-α in human breast cancer. Expression of FAP-α was characterized in primary tumour samples and in cell lines, along with the effects of FAP-α expression on *in vitro* growth, invasion, attachment and migration. Furthermore the potential interaction of FAP-α with other signalling pathways was investigated.

**Results:**

FAP-α was significantly increased in patients with poor outcome and survival. *In vitro* results showed that breast cancer cells over expressing FAP-α had increased growth ability and impaired migratory ability. The growth of MDA-MB-231 cells and the adhesion and invasion ability of both MCF-7 cells and MDA-MB-231 cells were not dramatically influenced by FAP-α expression. Over-expression of FAP-α resulted in a reduction of phosphorylated focal adhesion kinase (FAK) level in both cells cultured in normal media and serum-free media. An inhibitor to FAK restored the reduced motility ability of both MCF-7exp cells and MDA-MB-231exp cells and prevented the change in phosphorylated FAK levels. However, inhibitors to PI3K, ERK, PLCϒ, NWASP, ARP2/3, and ROCK had no influence this.

**Conclusions:**

FAP-α in significantly associated with poor outcome in patients with breast cancer*. In vitro*, FAP-α promotes proliferation and inhibits migration of breast cancer cells, potentially by regulating the FAK pathway. These results suggest FAP-α could be a target for future therapies.

## Background

Fibroblast Activation Protein alpha (FAP-α) or Seprase is a member of the serine integral membrane peptidases (SIMPs) family which also includes prolylendopeptidase, dipeptidyl peptidase IV (DPPIV or CD26), and dipeptidyl peptidase IIX. These peptidases are inducible, specific for proline-containing peptides, and are active on the cell surface [1,2]. Previous studies have demonstrated that FAP-α has an important role in development of cancers by modifying bioactive of substrate peptides and their cellular functions. However, the tissue distribution and function of FAP-α remains unclear.

FAP-α has been shown to be transiently expressed in certain normal fetal mesenchymal tissues, during wound healing and in reactive stroma responding to most o sarcomas and epithelial cancers including breast cancer, oesophageal cancer, colon cancer, pancreatic adenocarcinoma [3-6]. Normal adult tissues, haematopoietic cells as well as malignant epithelial cells are generally FAP-α -negative. However other studies have shown that FAP-α expression is not confined to stromal fibroblasts but that it is also expressed in some epithelial malignant cells. Kelly *et al.*[7] analyzed paraffin-embedded breast-cancer sections and revealed the expression of FAP-α in cancer cells. A study by Okada K *et al.*[8] showed that FAP-α immunoreactivity was recognized in both intestinal-type and diffuse type of gastric cancer accompanied with different levels of protein expression when detected by immunoblotting. FAP-α immunoreactivity was observed in some microinvasive and all invasive cervical carcinomas with various degrees of FAP-α -positive stromal cells [9]. Recently, it has been demonstrated that FAP-α is highly expressed on the surface of glioma cells, bone and soft tissue tumour cells [10,11].

There are also contradictive results about the function of FAP-α, in that it could act as both a tumour suppressor and tumour promoter. It has been observed that the expression of FAP-α decreased the tumourigenicity of mouse melanoma cells in animals and restored contact inhibition and growth factor dependence [12]. FAP-α has also been shown to suppress growth of NSCLC cells, accompanied by the increased expression of cell surface DPPIV [13]. Furthermore, increased stromal expression of FAP-α is shown to be associated with longer survival of breast cancer patients [3]. In contrast, it has also been shown that FAP-α can also act as a tumour promoter. Anti-sense suppression of FAP-α in human breast cancer cell lines MDA-MB-435 and MDA-MB-436, which normally express FAP-α rendered these cells sensitive to serum starvation, whilst high levels of FAP-α expression were less dependent on exogenous serum factors for growth and gained independence from normal growth regulatory controls [14]. The human breast cancer cell line MDA-MB-231 expressing FAP-α grew more rapidly and was produced highly vascular tumours *in vivo*[15]. Mice inoculated with FAP-transfected HEK293 cells were two to four times more likely to develop tumours compared with vector-transfected HEK293 controls, with a 10- to 40-fold enhancement in tumour growth [16]. Antibody abrogation of FAP-α enzymatic activity by site-directed mutagenesis of FAP-α was shown to result in a significant reduction in FAP-driven tumour growth *in vivo*[17].

A recent investigation suggested that FAP-α promoted tumour growth and invasion of breast cancer cells might be through non-enzymatic functions. Huang *et al.*[18] introduced different inhibitors of prolyl peptidases including Val-boroPro (talabostat); Glu-boroPro (PT-630); or 1-[[(3-hydroxy-1-adamantyl)amino]acetyl]-2-cyano-(S)-pyrrolidine (LAF-237) to investigate the function of FAP-α on breast cancer cells in a SCID mice model. Their results showed that PT-630 and LAF-237 did not slow the growth of tumours produced by any of the three breast cancer cell lines expressing FAP-α. Talabostat slightly decreased the growth rates of the FAP-α -expressing tumours but the growth retardation was likely not related to the inhibition of FAP-α or the related post-prolyl peptidase dipeptidyl peptidase IV. Breast cancer cells expressing a catalytically inactive mutant of FAP-α (FAPS624A) also produced tumours that grew rapidly [18]. In another study, the over-expression of FAP-α in the human hepatic stellate cell (HSC) cell line LX-2 increased cell adhesion, migration and invasion. However the proteolytic activity of FAP-α was not necessary for these functions [19]. These findings imply that in addition to its enzymatic functions, FAP-α might have important non-enzymatic functions involved in regulating the development and spread of cancer cells.

Therefore, in this study we analyzed the function of FAP-α in breast cancer cells with the intention to explore the non-enzymatic function of FAP-α. Our hypothesis is that as a membrane protein, FAP-α might participate in the regulation of other membrane molecules or signaling pathways by which exert its influence on tumour cells.

## Methods

### Materials and cell lines

Human breast cancer cells, MCF7 and MDA-MB-231 were from the ATCC (American Type Cell Collection, Manassas, VA, USA). Fresh frozen human breast tissues were collected from University Hospital of Wales under the approval of the local ethical committee, obtained immediately after surgery and stored at -80°C until used.

Antibodies to human FAP-α (sc-100528 and ab5066), FAK (sc-1680) and pFAK (sc-11765-R were from Santa-Cruz Biotechnologies, Inc. (Santa Cruz, CA, USA or Abcam, Cambridge, UK). ROCK inhibitor was from Santa-Cruz Biotechnologies, Inc. (Santa Cruz, CA, USA), ERK inhibitor, Wortmannin, and Wiskostatin were from Calbiochem (Nottingham, UK). Matrigel (reconstituted basement membrane) was purchased from Collaborative Research Products (Bedford, MA, USA). Transwell plates equipped with a porous insert (pore size 8 μm) were from Becton Dickinson Labware (Oxford, UK). RT-PCR reagents and plasmid extraction kits were from Sigma (St. Louis, MO, USA).

### Construction of expression vector of human FAP-α and transfection of breast cancer cells

Touch-down PCR was used to generate the cDNA of FAP-α from human prostate tissues with primers 5’-TTAGTCTGACAAAGAGAAACACTG and 5’-ATGAAGACTTGGGTAAAAATCG. The cDNA of FAP-α was subsequently cloned into a pEF6/V5-His vector (Invitrogen, Paisley, Scotland, UK). The new plasmid, named pEF6/V5- FAP was amplified in *E. coli* and verified by PCR reaction by using a pair of different primers 5’-AGAGCTTTAGCAATCTGTGC and 5’-TCCCTTGCTAATTCAAGTGT.

Breast cancer cells MCF7 and MDA-MB-231 were cultured in DMEM media. The cells were transfected with plasmid pEF6/V5- FAP-α by electroporation. Following selection of transfected cells with blasticidin (used at 5 μg/ml) and verification by PCR, the stably transfected cells were established: FAP-α over-expression cells MCF7exp and MDA-MB-231 exp, plasmid only control cells MCR7pef and MDA-MB-231pef and the wild type cells MCF7wt and MDA-MB-231wt. The transfected cells thus created were always kept in a maintenance medium which contained 0.5 μg/ml blasticidin. Pooled populations of genetically manipulated cells from multiple clones were used in the subsequent studies.

### In vitro cell function including cell growth, adhesion, invasion, and migration assay

Cell growth assay: cells were plated into 96-well plated at 2,000 cells/well. Cells were fixed in 10% formaldehyde on the day of plating, and the day3 and day 5 subsequently. 0.5% crystal violet (w/v) was used to stain cells. Following washing, the stained crystal violet was dissolved with 10% (v/v) acetic acid and the absorbance was determined at a wavelength of 540 nm using an ELx800 spectrophotometer (Bio-Tek, ELx800). Absorbance represents the cell number.

Adhesion assay: a 96-well plate was pre-coated with 5 μg of Matrigel and allowed to dry overnight. Following rehydration with serum-free media, 20,000 cells were seeded into each well. After 40 min of incubation, non-adherent cells were washed off using BSS buffer. The remaining cells were fixed with 4% formalin and stained with 0.5% crystal violet. The number of adherent cells was then counted under microscopy.

Invasion assay: transwell inserts (upper chamber) with 8 μm pore size were coated with 50 μg of Matrigel (Collaborative Research Products, Bedford, Massachusetts, USA) and air-dried. Following rehydration with serum-free media, cells were seeded at a density of 30,000 per insert. After 3 day’s incubation, cells that had migrated through the matrix and adhered to the other side of the insert were fixed in 4% formalin, stained with 0.5% (weight/volume) crystal violet, and counted under a microscope.

Migration/wounding assay: cells were seeded at a density of 250,000 per well into a 24-well plate and allowed to reach confluence by overnight culture. The monolayer of cells was then scraped with a fine gauge needle to create a wound of approximately 200 μm. The movement of cells to close the wound was recorded for 4 hours. The movement of cells were analyzed by tracking cell boundary, for each frame in a series, using the Optimas 6.0 motion analysis (Meyer Instruments, Houston, Texas).

### Electric Cell-substrate Impedance Sensing (ECIS) based cell adhesion and motility assay

Electric Cell-substrate Impedance Sensing (ECIS, Applied Biophysics Inc, Troy, NY, USA) instrument ECIS Zθ (Theta) was also used to record both cell adhesion and migration abilities which were shown here as the changes of resistance. 96W1E arrays were incubated with complete medium for 1 hour. 50,000 cells of breast cancer cells were seeded into each well. The electric changes were continuously monitored for up to 24 hr while an electric wounding was performed after 6 hours. Multiple conditions of frequency 1000 Hz, 2000Hz, 4,000 Hz, and 8,000 Hz were used to screen the nature of resistance changes.

### Influence of inhibitors of signalling pathway on adhesion and migration of breast cancer cells by ECIS assay

In order to explore the potential crosstalk of FAP-α and other adhesion and migration associated signalling pathway. We introduced inhibitors of FAK, ROCK, PLC-γ, and PI3K pathway in ECIS based cell adhesion and motility assay. 50,000 cells of breast cancer cells were suspended in 200 ul media with inhibitors of FAK, ROCK, PLC-γ, and PI3K respectively and the final concentration was 100 nm. The electric changes were continuously monitored for up to 24 hr under multiple condition of frequency while an electric wounding was performed after 6 hours.

### Flow cytometric analysis of in breast cancer cells

In this study, we utilised the Vybrant® Apoptosis Assay Kit (Invitrogen, Inc., Paisley, UK) to perform the apoptosis assay. Cells including those suspended in the culture medium were harvested and washed in cold BSS buffer. After centrifugation, the cell pellet was resuspended in 1X annexin-binding buffer. Determine the cell density and dilute in 1X annexin-binding buffer to about 1 × 10^6^ cells/ml. 5 μl of FITC annexin V and 1 μl of the PI working solution (100 μg/ml) were added to each 100 μl of cell suspension and incubated at room temperature for 5 min. After the incubation, 400 μl of 1X annexin-binding buffer was added, mixed gently and stored on ice. Cells were analyzed using the Partec CyFlow® SL flow cytometer and FlowMax software package (Partec GmbH, Munster, Germany), measuring the fluorescence emission at 530 nm and >575 nm.

### Immunofluorescence staining in breast cancer cells

20,000 cells were seeded in each well of a 16-well chamber and cultured overnight. Then cells were fixed in 100% ethanol for 30 minutes. After blocked in a 10% horse serum solution, cells were incubated with primary antibodies overnight and were incubated for 30 min in the secondary FITC- and TRITC conjugated antibodies. Following extensive washings, the slides were mounted using Fluorsave™ mounting media (Calbiochem, Nottingham, UK) and allowed to harden overnight in the refrigerator before being examined. Slides were examined using an Olympus fluorescence microscope and photographed using a Hamamatsu digital camera. The images were documented using the Cellysis software (Olympus, Bristol, England, UK).

### Western blotting and Immunoprecipitation

To detect the expression level of FAP-α in breast cancer cell lines, confluent cells were pelleted and then lysed using a lysis buffer containing 2.4 mg/ml Tris, 4.4 mg/ml NaCl, 5 mg/ml sodium deoxycholate, 20 μg/ml sodium azide, 1.5% Triton, 100 μg/ml PMSF, 1 μg/ml leupeptin, and 1 μg/ml aprotinin, for 45 min at 4°C. After lysis and centrifugation at 13,000 rpm for 15 min, protein concentrations for each sample were measured using an improved Lowary assay (DC Protein Assay kit, Bio-Rad). The samples were adjusted to equal concentrations with sample buffer and then boiled at 100°C for 5 min, before separated on a 10% polyacrylamide gel. Following electrophoresis, these separated proteins were transferred onto nitrocellulose sheets and blocked in 10% skimmed milk (w/v in TBS) for overnight. The membranes were then probed with the anti-FAP-α, anti-FAK antibodies, and anti-GAPDH antibody as internal control, followed by a peroxidase-conjugated secondary antibody. Protein bands were visualised using an ECL system (Amersham, UK), and photographed using an UVITech imager (UVITech, Inc). The proteins obtained from breast cancer cells were immunoprecipitated with 10 μl of anti-FAP-α and anti-FAK antibodies for 2 h at 4°C followed by the addition of 20 μl of protein A/G-agarose beads overnight at 4°C. The resultant pellet was subjected to SDS-PAGE and Western blotting by antibodies against the FAP-α and phosphatised FAK.

### Human breast tissues

A total of 133 breast samples were obtained from breast cancer patients (106 breast cancer tissues and 27 associated background or related normal tissue). The anonymised breast tissue samples were obtained within the guidelines of the appropriate ethics committee (Bro Taf Health Authority 01/4303 and 01/4046). Informed patient consent was not applicable in this instance (as stated in the Human Tissue Act 2004, UK). The pathologist verified normal background and cancer specimens, and it was confirmed that the background samples were free from tumour deposit. These tissues after mastectomy were immediately frozen in liquid nitrogen.

### Real-time quantitative Polymerase Chain Reaction (Q-PCR)

The assay was based on the Amplifluor system. It was used to detect and quantify transcript copy number of FAP-α in tumour and background samples. Primers were designed by Beacon Designer software, which included complementary sequence to universal Z probe (Intergen, Inc.). Each reaction contains 1 pmol reverse primer (which has the Z sequence), 10 pmol of FAM-tagged universal Z probe (Intergen, Inc.) and cDNA (equivalent to 50 ng RNA). Sample cDNA was amplified and quantified over a large number of shorter cycles using an iCyclerIQ thermal cycler and detection software (BioRad laboratories, Hammelhempstead, UK) under the following conditions: an initial 5 minute 94°C period followed by 60 cycles of 94°C for 10 seconds, 55°C for 15 seconds and 72°C for 20 seconds. Detection of GAPDH copy number within these samples was later used to allow further standardisation and normalisation of the samples.

### Statistical Analysis

All of the results are expressed as the means ± S.E. Cell growth, wounding or migration, adhesion, and invasion formation was analyzed using a Student's t test on log normalized data or Mann Witney for patient tissues (where required).

## Results

### Expression of FAP-α in breast tumour is correlated with patient prognosis and survival

**Figure 1:**
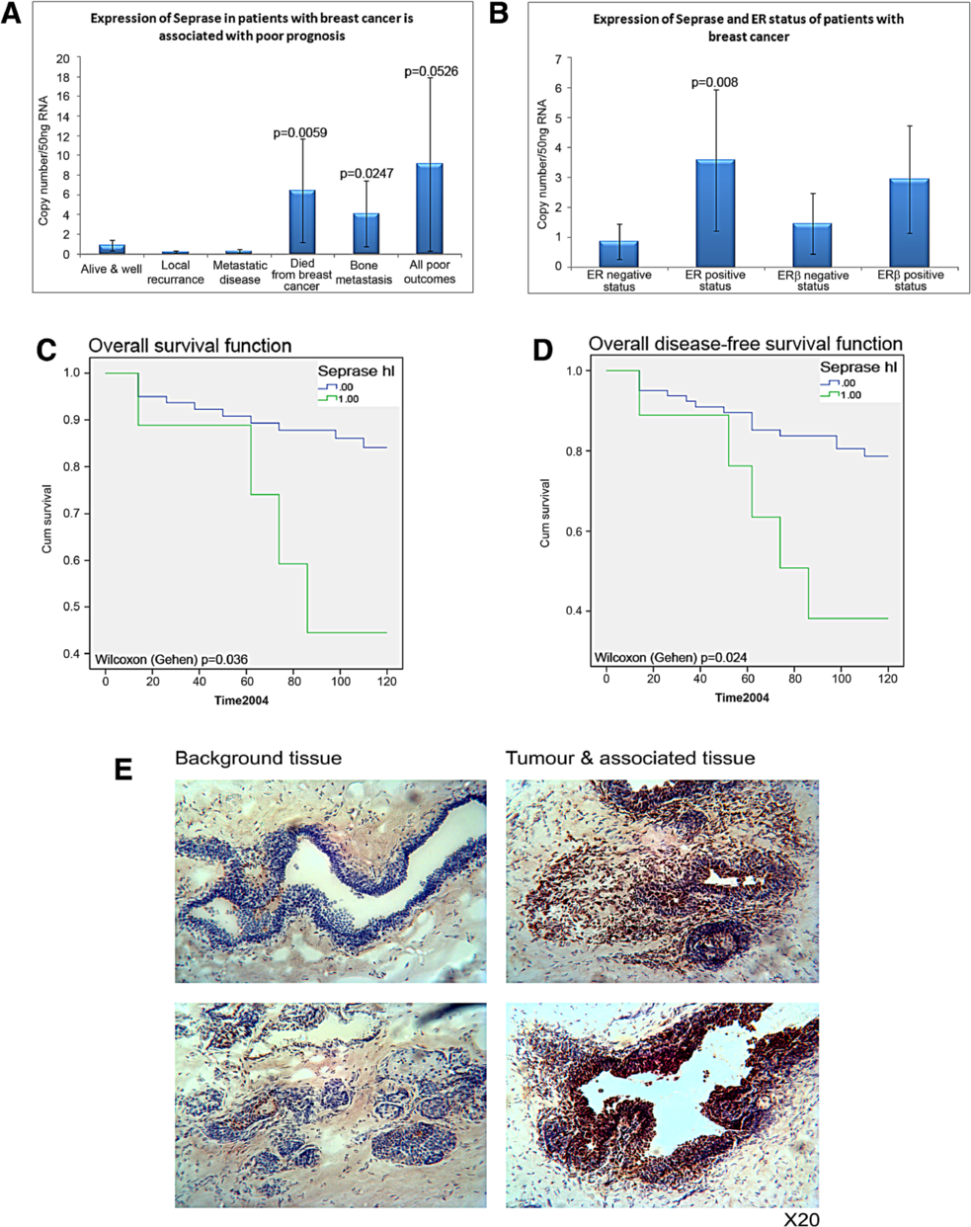
**Analysis of human breast cancer and background tissues. (A)** Q-PCR revealed a significant increase in FAP-α expression with poor patient outcome. **(B)** Expression was also increased in ER positive and ERβ positive patients. **(C)** Kaplan-Meier survival curves showing overall survival analysis of patients expressing FAP-α. **(D)** Kaplan-Meier survival curves showing disease-free survival analysis of patients with breast cancer. **(E)** Immunohistochemical staining of FAP-α in background breast tissues (left) and tumour/associated tissues (right).

Analysis carried out using Q-PCR (using CK-19 to normalise) revealed that patients with poor outcome had the highest levels of FAP-α (Figure 1A). Patients who remained alive and well had significantly lower levels of FAP-α than those who had died from breast cancer or those who had poor outcomes in general (normalised transcript copy number/50 ng RNA was alive and well 0.878 ± 0.533; median value <0.001: died from breast cancer 6.44 ± 5.27; median value 0.1: all poor outcomes (recurrence, metastasis, death) 4.11 ± 3.31; median value 0.02, p = 0.0059 and p = 0.0247 respectively). Lower levels were also evident in those patients who had bone metastasis (9.1 ± 8.8; median value 0.02, p = 0.052). FAP-α was also significantly increased in ER positive and ERβ positive tumours (Figure 1B). When long-term survival was analysed using Kaplan-Meier survival curves, patients with high levels of FAP-α transcript had a significantly shorter survival than patients with low levels of FAP-α (p = 0.036); High levels of FAP-α, mean survival 98.222 ± 17 months (64.853-131.591 months, 95% CI) versus low levels of FAP-α, 137.587 ± 4.84 months (128.587-147.086 months, 95% CI, cut-offs as previously determined [20]). This was also seen in patients who had remained disease free, who had lower levels of FAP-α (p = 0.024); High levels of FAP-α, mean survival 90.73 ± 16.8717 months (58.447-121.983 months, 95% CI) versus low levels of FAP-α, 132.313 ± 5.27 months (121.963-142.642 months, 95% CI). Immunohistochemical staining showed a corresponding increase in protein levels of FAP-α in tumour tissues when compared to background tissue (Figure 1E). The data for the patient cohort is summarised in Table 1.

**Table 1:** Patient population data of samples included in the analyses

**Tissue Type**							
	Background (32)	Tumour (120)					
		Tumour Grade					
			Grade 1 (22)	Grade 2 (40)	Grade 3 (15)	Unknown (43)	
		NPI					
			NPI 1 (43)	NPI 2 (37)	NPI 3(15)	Unknown (5)	
		TNM					
			TNM 1 (22)	TNM 2 (37)	TNM 3 (7)	TNM 4 (4)	Unknown (48)
		Histology					
			Ductal (94)	Lobular (13)	Other (13)		
		Outcome					
			Disease free (86)	Metastatic disease (6)	Local recurrence (5)	Death from breast cancer (16)	All poor outcomes (27)

### Expression of FAP-α in breast cancer cell lines

**Figure 2:**
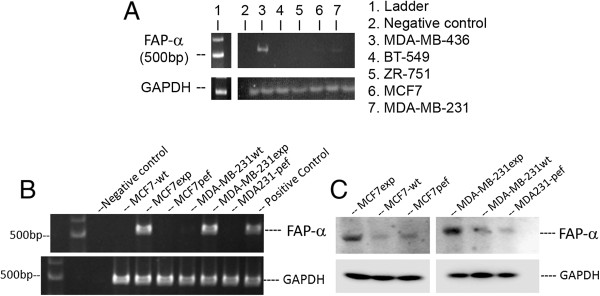
**Expression of FAP-α in human breast cancer cell lines. (A)** RT-PCR showing the lack of expression of FAP-α in MCF7 and MDA-MB-231 human breast cancer cell lines. **(B)** Cells transfected with a FAP-α expression plasmid. GAPDH was used as a control. **(C)** Western blotting showing successful expression of FAP-α in both cell lines.

We analysed the expression pattern of FAP-α in MCF7 and MDA-MB-231 breast cancer cell lines. When using RT-PCR, we found no transcript of FAP-α mRNA in the breast cancer cell lines (Figure 2A). After transfection with plasmid pEF6/V5-FAP-α, transcription of FAP-α mRNA could be amplified and an increased expression of FAP-α was also detected in MCF7exp and MDA-MB-231exp cells (Figure 1B). GAPDH was used as a standard control. Western blotting for the FAP-α protein showed successful protein expression in both MCF7 and MDA-MB-231 cells (Figure 1C).

### Over-expression of FAP-α promotes growth of MCF-7 cells and has no effect on apoptosis

**Figure 3:**
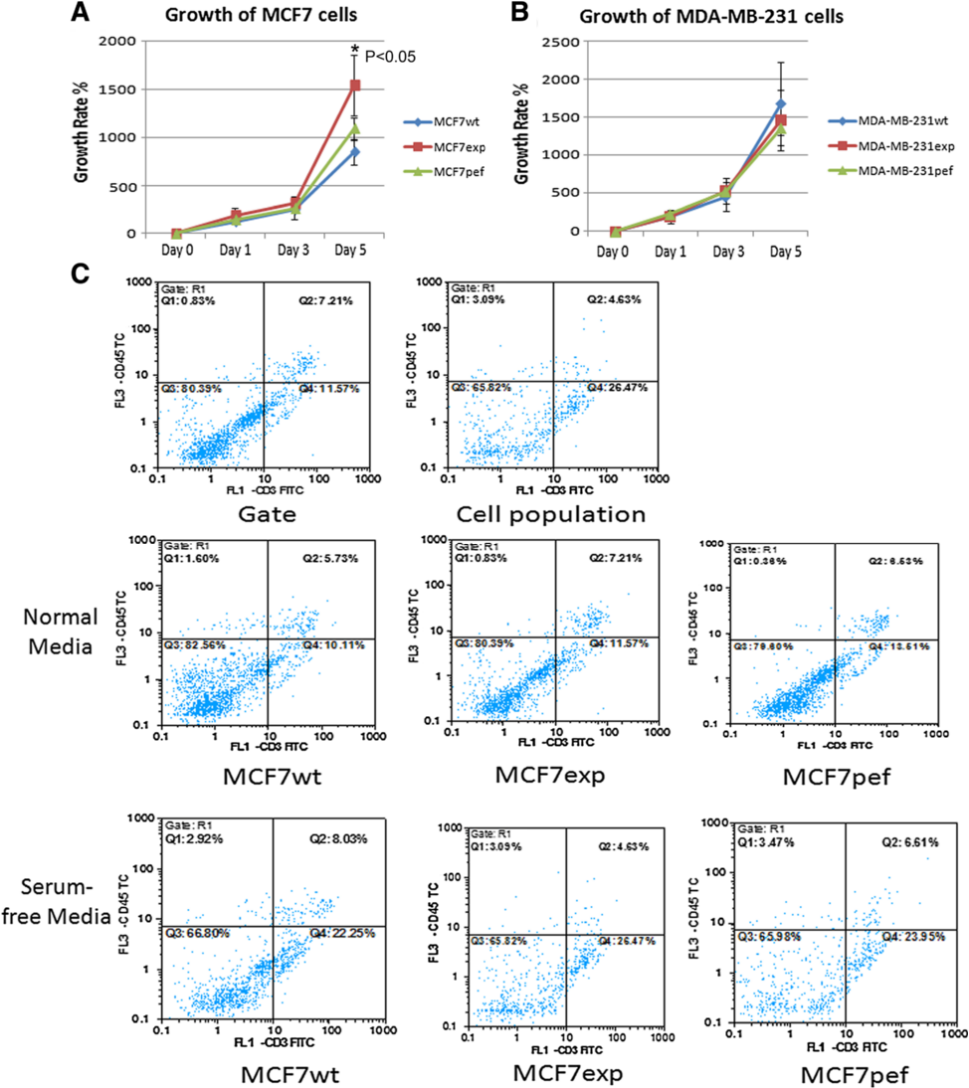
**Growth of human breast cancer cells overexpressing FAP-α. (A)** There was an increase in growth of MCF7exp cells compared to the controls (n = 8). **(B)** No equivalent increase was observed in MDA-MB-231 cells. **(C)** Apoptosis was not reduced in MCF7exp cells.

In the *in vitro* cell growth assay, it was observed that a significantly higher rate of growth was achieved in FAP-α transfected MCF7 cells (Figure 3A), but that there was little change in MDA-MB-231 breast cancer cell growth (Figure 3B). In order to ascertain whether apoptosis in the cells was reduced, we used flow cytometry to analyse the relative expression of Annexin V. The results showed that the rate of apoptosis in MCF7wt, MCF7pef, and MCF7exp were 9.03%, 6.61%, 4.63% respectively in serum free medium and those in cells cultured in normal media were 5.73%, 6.53%, and 7.21%. There was no significant difference between cells (Figure 3C).

### Over-expression of FAP-α impairs human breast cancer cell migration

**Figure 4:**
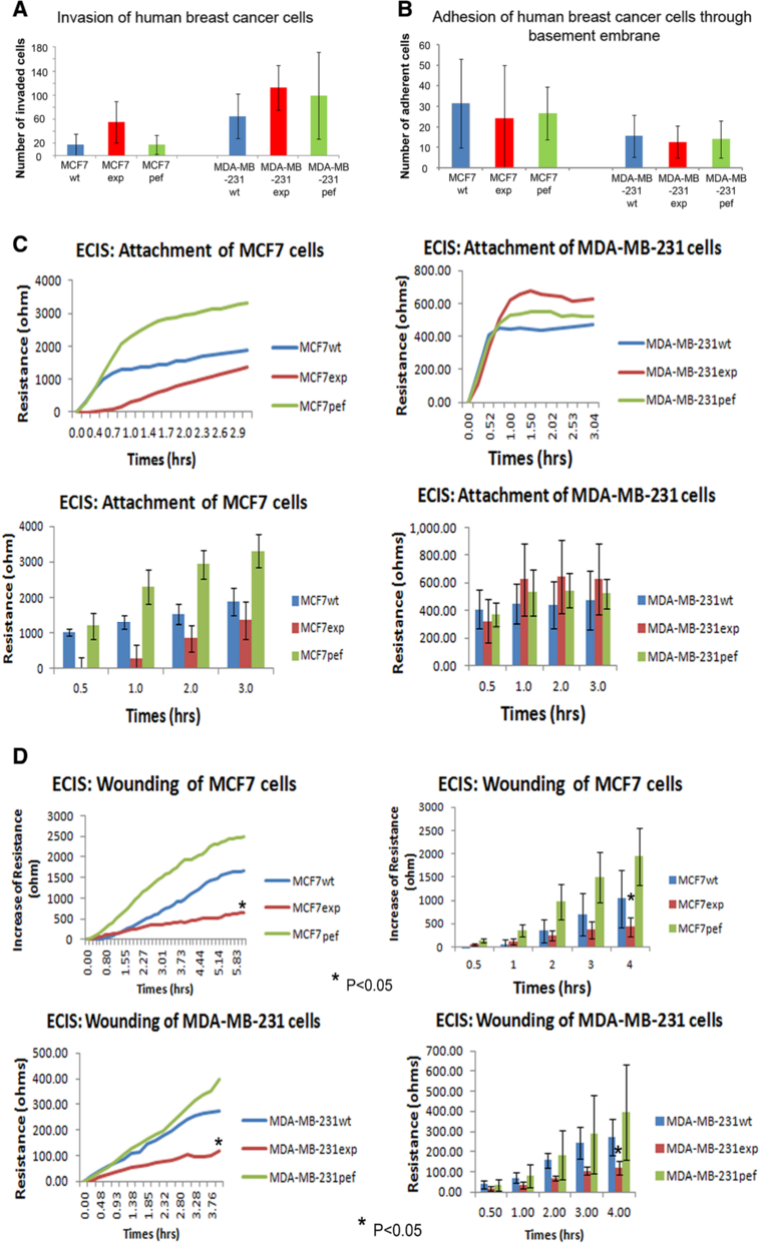
**Effect on FAP-α overexpression and cellular behaviour. (A)** Increased invasion was observed in both MCF7 and MDA-MB-231 cells Overexpressing FAP-α (n = 8). **(B)** Adhesion to basement membrane showed that MCF7exp cells exhibited lower adhesion but that MDA-MB-231exp cells were more adhesive (n = 8). **(C, top)** ECIS experiments showed that MCF7exp cell showed reduced attachment after 2 hour, whereas MDA-MB-231exp attachment was increased **(C, bottom)**. **(D)** In the migration phase, motility of both MCF7exp cells and MDA-MB-231exp cells was dramatically reduced compared to the wild type and control cells (n = 16).

Using a matrigel based *in vitro* invasion assay, it was found that MCF7exp and MDA-MB-231exp cells had increased invasion ability compared with those of the wild type and control cells although this did not reach significance (MCF7wt, MCF7pef, MCF7exp, MDA-MB-231wt, MDA-MB-231pef, and MDA-MB-231exp cells were 17.8 ± 17.3, 17.4 ± 15.7, 55.3 ± 34.8, 65.2 ± 36.9, 99.0 ± 72.2, and 112.6 ± 36.8 respectively, P > 0.05) (Figure 4A). When we examined any effect on cell adhesion to basement membrane, it was seen that MCF7exp cells exhibited lower adhesion but that MDA-MB-231exp cells were more adhesive compared with those of the wild type and control cells, although this did not achieve significance (MCF7wt, MCF7pef, MCF7exp, MDA-MB-231wt, MDA-MB-231pef, and MDA-MB-231exp cells were 31.3 ± 20.6, 24.3 ± 12.4, 26.7 ± 31.5, 12.7 ± 18.8, 15.5 ± 15.6, and 14.0 ± 14.4 respectively, P > 0.05) (Figure 4B). This may be due to the different aggressive behaviour of the cell lines, as MDA-MB-231 cells are intrinsically more invasive.To investigate the impact of FAP-α on migration of cells, we used the ECIS based wounding assay. This enabled a detailed analysis of the attachment and migration of the cells in two spate phases. ECIS enables us to firstly measure the rate of attachment of the cells to the substratum; the faster the resistance increases, the greater the rate of attachment, and secondly by enabling us to measure migration of cells into a wound created in the confluent cell monolayer after the addition of a high voltage shock. The reduced resistance associated with the “wound” is then reversed as cells move into the cleared area, the resistance plateauing when the cleared space is filled. In the adhesion phase of ECIS, MCF7exp cell showed reduced attachment after 2 hours, whereas MDA-MB-231exp attachment was increased, compared to control cells (Figure 4B) (p < 0.05, n = 8). The confluent cell monolayer was then wounded and the experiment resumed to examine changes in migration. In the migration phase, the results showed that the motility of both MCF7exp cells and MDA-MB-231exp cells was dramatically reduced compared to the wild type and control cells (P < 0.05) (Figure 4D).

### FAK inhibitor could restore the impaired motility ability of breast cancer cells

**Figure 5:**
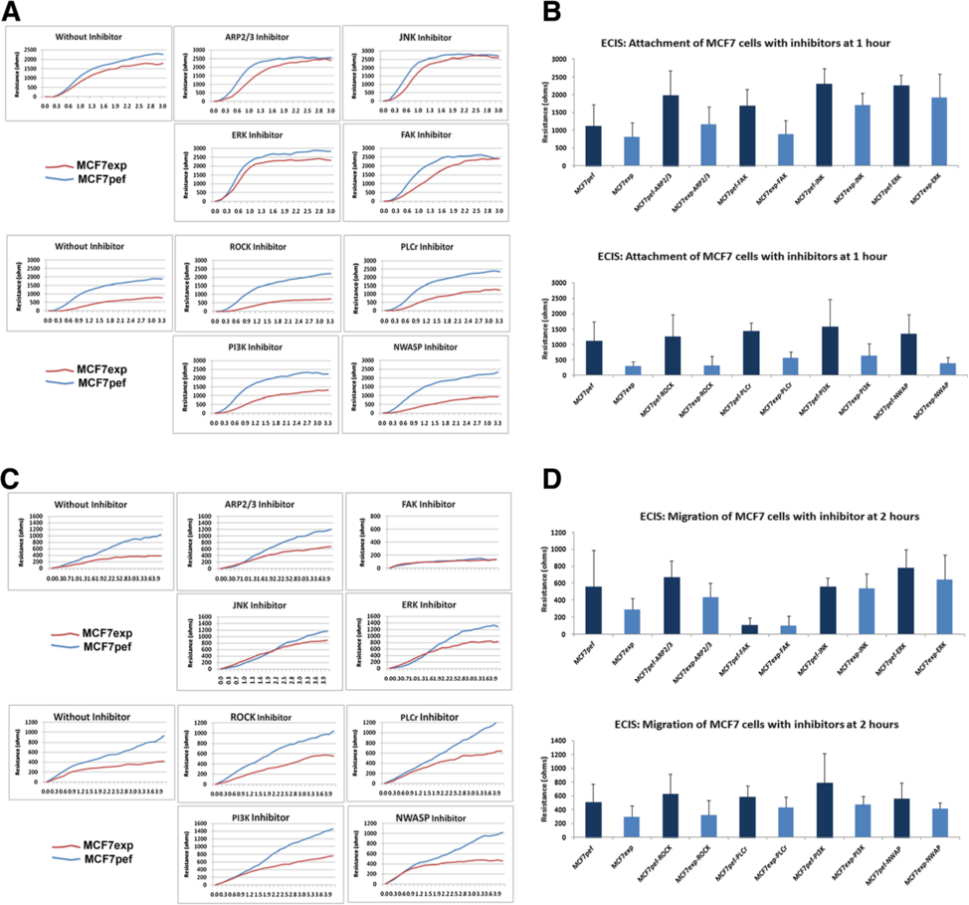
**Effect of small inhibitors on the impaired motility ability of MCF7 breast cancer cells.** A panel of small inhibitors linked to motility was screened, with only the FAK inhibitor having a substantial effect. ECIS was used to assess the effect of these inhibitors on cell migration/motility. Again, we looked at the two phases of the experiment, attachment and migration. **(A and B)** There was little difference in attachment upon addition of the small inhibitors to MCF7exp cells (n-16). **(C and D)** The FAK inhibitor was seen to partially restore the inhibitory effect of FAP-α on the motility of MCF7exp cells (n = 16).

**Figure 6:**
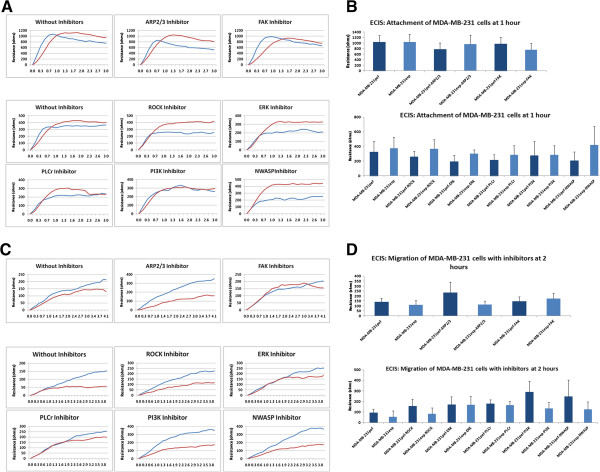
**Effect of small inhibitors on the impaired motility ability of MDA-MB-231 breast cancer cells. (A and B)** As for MCF-7 cells, there was little difference in after the addition of the inhibitors on MDA-MB-231exp attachment (n = 16). **(C and D)** The FAK inhibitor was able to partially restore the inhibitory effect of FAP-α expression in MDA-MB-231 cells (n = 16) **(D)**.

In searching for the potential pathway(s) that may be responsible for the impact of FAP-α expression on breast cancer cells, we screened a panel of small molecule inhibitors to some of the key signalling pathways that are linked to cell motility. They included the ROCK inhibitor, JNK inhibitor, PI3K inhibitor, FAK inhibitor and ERK inhibitor. Using ECIS (n = 16 for each experiment), we assessed the effect of these inhibitors on cell migration/motility. Again, we looked at the two phases of the experiment, attachment and migration. There was little difference in attachment upon addition of the small inhibitors to MCF7exp cells (not shown) but a small difference with the FAK inhibitor (Figure 5A and B). Only the FAK inhibitor was seen to partially restore the inhibitory effect of FAP-α on the motility of MCF7exp cells (Figure 5C and D). Again, there was little difference in after the addition of the inhibitors on MDA-MB-231exp attachment (Figure 6A and B). The FAK inhibitor was able to partially restore the inhibitory effect of FAP-α expression in MDA-MB-231 cells (Figure 6C and D), as in the MCF7 cells. ERK inhibitor had only a marginal effect on the motility of the control breast cancer cells however it reversed the inhibition of motility in MDA-MB-231exp cells to that of the control. This was more evident at the early phase (within 2 hours after wounding) (Figure 6D).

### Over-expression of FAP-α accompanied with a reduction of phosphorylated FAK

**Figure 7:**
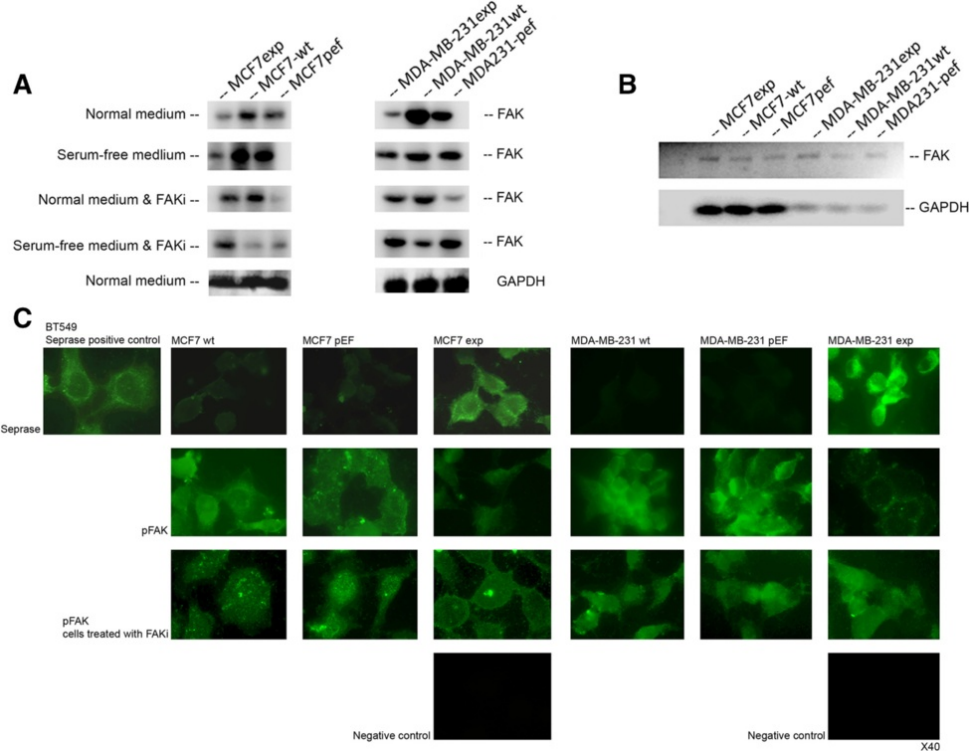
**Effect of FAP-α overexpression on FAK phosphorylation. (A)** over-expression of FAP-α in MCF7exp cells and MDA-MB-231exp cells accompanied a decrease in phosphorylated FAK (pFAK) when cultured in both normal media and serum-free media which was restored on addition of FAKi. **(B)** over-expression of FAP-α in MCF7exp and MDA-MB-231exp cells had no influence on the transcription of FAK compared with those of wild type and control cells. **(C)** Immunofluorescence staining of FAP-α and FAK in both MCF7 and MDA-MB-231 cells showed that the over-expression of FAP-α accompanied a reduction in the fluorescence intensity of phosphorylated FAK which was partially restored using FAKi. The human breast cancer cell line BT549 was used as a positive control for endogenous FAP-α expression.

To investigate the potential crosstalk of FAP-α and the FAK pathway, we analyzed the proteins expression of FAP-α and the phosphorylation status of FAK. As shown in Figure 7A, over-expression of FAP-α in MCF7exp cells and MDA-MB-231exp cells accompanied a decrease in phosphorylated FAK (pFAK) when cultured in both normal media and serum-free media. Addition of the FAK inhibitor (FAKi) could restore the levels of FAK phosphorylation in both MCF7exp cells and MDA-MB-231exp cells (Figure 7A). Furthermore, we analyzed the expression of FAK in all genetically modified cells. The results showed that the over-expression of FAP-α in MCF7exp and MDA-MB-231exp cells had no influence on the transcription of FAK compared with those of wild type and control cells (Figure 7B).Immunofluorescent staining of FAP-α and FAK in both MCF7 and MDA-MB-231 cells showed that the over-expression of FAP-α accompanied a reduction in the fluorescence intensity of phosphorylated FAK (Figure 7C, second row). In addition, the inhibitor of FAK could partially restore the expression of phosphorylated FAK (Figure 7C, bottom row). The human breast cancer cell line BT549 was used as a positive control for endogenous FAP-α expression.

## Discussion

Fibroblast Activation Protein alpha (FAP-α) is an integral membrane serine peptidase. It has been shown that it plays an important role in tumour proliferation, migration, invasion and angiogenesis. Recent studies have provided convincing evidence that targeting FAP-α is a promising method in both diagnosis and treatment of cancer. A combination of FAP-α with other serum markers such as CEA, CYFRA 21–1, OPN, ferritin, and anti-p53 had comparable sensitivity with faecal immunochemical testing (FIT) for the early detection of colorectal cancer [21]. Immunotherapy targeting FAP-α could inhibit tumour growth and increases survival in a murine colon cancer model. A DNA vaccine directed against FAP-α could significantly suppressed primary tumour and pulmonary metastases through CD8+ T-cell-mediated killing in tumour-bearing mice [22]. An antibody-maytansinoid conjugate, monoclonal antibody (mAb) FAP5-DM1 targeted at a shared epitope of human, mouse, and cynomolgus monkey FAP, could induced long-lasting inhibition of tumour growth and complete regressions in xenograft models of lung, pancreas, and head and neck cancers with no signs of intolerability [23]. The clinical impact of FAP-α was also tested using Val-boroPro (Talabostat), the first clinical inhibitor of FAP-α enzymatic activity, in a phase II study of patients with metastatic colorectal cancer. The results showed that minimal clinical activity was also observed in patients with previously treated metastatic colorectal cancer [24]. Our data has shown that in patients with breast cancer, FAP-α is significantly over-expressed in those with poor prognosis and is inversely related to both overall and disease-free survival. However, until now, there is still no conclusion as to the relation between FAP-α expression and function.

Active FAP-α is a 170 kDa homodimer that contains two N-glycosylated 97 kDa subunits. The FAP-α monomer has C-terminal catalytic domains of serine proteases, a hydrophobic transmembrane segment and a short cytoplasmic tail [1,25]. Studies of the mouse homologue have shown that alternative splicing and 3 distinct FAP-α splice variants had been detected in tissues [26]. An alternative spliced FAP-α was later identified in the human melanoma cell line LOX which encodes a truncated isoform which encodes a 239 amino acid polypeptide with a molecular weight of 27 kDa that precisely overlaps the carboxylterminal catalytic region of the wild type FAP-α [27]. In another study using a generated soluble recombinant FAP-α, it was found that in the presence of putative EDTA sensitive activators, FAP-α was converted into 70 kDa to 50 kDa shortened forms [28].

In our study, a 549 bp product of FAP-α could be amplified in both MCF7exp and MDA-MB-231exp cells while the western-blotting assay of FAP-α protein expression in breast cancer cells showed that a 30 kDa truncate was detected instead of the 97 kDa monomer and 170 kDa dimer. We presumed that it might be caused by the degradation of FAP-α protein or the use of antibodies that recognize, with varying affinity, different epitopes exhibited by FAP-α.

Some studies have suggested that FAP-α is expressed by stromal cells rather than cancer cells in epithelial malignant diseases; however other studies have demonstrated that FAP-α is also expressed in epithelial malignant cells such as breast cancer, gastric carcinoma, and cervical carcinoma [7,8]. In our study, we analyzed the expression of FAP-α by PCR reaction and western-blot assay. The results showed that there was no amplification of FAP-α mRNA in both MCF7 and MDA-MB-231 wild type cells but a weak expression of FAP-α protein were detected in these cells by immunoblotting. Moreover, FAP-α was detected in BT-549 breast cancer cells. We tentatively suggest that the negative result in the earlier studies might be due to the comparative difference in staining intensity between the high expressions in stromal cells compared to the weaker expression of FAP-α in cancer cells.

Over-expression of FAP-α in fibroblasts and paracytes of cancer cells promotes tumour growth, invasion and metastasis by directly remodelling extracellular matrix and targeting fibroblast activation protein could inhibit tumour stromagenesis and growth. Furthermore, some clinical data has implied that patients with over-expression of FAP-α identified from stroma had a significantly poorer outcome [4,29]. Again, there are also contradictive results regarding the function of FAP-α in that it could act as both a tumour suppressor and tumour promoter. Our results showed that overexpression of FAP-α could promote the growth of MCF7 cells, while no significant difference in proliferation was observed in MDA-MB-231exp cells compared with wild type and control cells. A previous study by Huang *et al.*[15] also d that MDA-MB-231 cells genetically expressing FAP-α grew at the same rate as control cells without the expression of FAP-α *in vitro*. However, when using a mouse model *in vivo*, MDA-MB-231 cells expressing FAP-α grew more rapidly and were highly vascular compared to control cells *in vivo*[15]. These results indicated that FAP-α could act as a tumour promoter and this tumour promotion effect was more noticeable *in vivo*.

The process of metastasis and invasion of tumour cells requires these cells to alter their ability to adhere or detach to both stromal cells and the extracellular matrix (ECM). Membrane Integrins play a crucial role in many aspects of tumour initiation and progression [30,31]. Focal adhesion kinase (FAK), an intracellular tyrosine kinase recruited to the sites of integrin clustering or focal adhesions, functions as a major mediator of signal transduction by cell surface receptors including integrins, growth factor and cytokine receptors progression [31-33]. FAK has been shown to play a key role in the regulation of cell adhesion, migration, and invasion [33-35]. Our study discovered that over-expression of FAP-α resulted in decreasing adhesion and migration ability of MCF7 and MDA-MB-231 cells. However, an inhibitor of FAK could restore the reduced motility ability of both MCF-7exp cells and MDA-MB-231exp cells, while inhibitors to PI3K, ERK and ROCK had no influence on it. Furthermore, using an immunoprecipitation assay and protein samples extracted from cells cultured in normal media and serum-free media, we found that the over-expression of FAP-α was associated with a decrease in phosphorylated FAK protein under both normal culture and serum-free culture condition. Moreover, an inhibitor to FAK could eliminate this difference in phosphorylated FAK in all the MCF-7 and MDA-MB-231 cell lines. This implies that FAP-α might form a complex with the FAK protein and so reduce its phosphorylation, which thus results in reduction of adhesion and motility ability. Earlier work had shown that cells expressing FAP-α exhibited a decrease in FAK phosphorylation in a murine model [36]. It has been previously shown that depletion of FAP-α-expressing stromal cells can impair the growth of immunogenic tumours via a lymphocyte dependent mechanism and that inhibition of FAP-α using extracellular competitive inhibitors of dipeptidyl peptidases have been shown to contribute to impaired epithelial cancer growth via a FAP-α dependant mechanism [36-39]. Little is known about intracellular post-proline cleaving enzymes such as FAP-α in the context of tumour behaviour and growth compared to other extracellular proteases [40].

## Conclusions

In conclusion, we report that FAP-α expression in patients with breast cancer is increased with poor prognosis and patient survival. In vitro data shows that this increased expression leads to decreased adhesion and migration of human breast cancer cells. Moreover, this was associated with increased growth. This effect appeared to be integrated with the phosphorylation status of FAK, which could be part of the control pathway by which FAP-α effects change in cancer cells. Further work is necessary to dissect the pathway in which FAP-α is involved.

## Abbreviations

FAP-α: Fibroblast Activation Protein-α; FAK: Focal adhesion kinase; PIK3: Phosphoinositide 3-kinase; ERK: Extracellular signal-regulated kinase; PLCϒ: Phospholipase C -gamma; NWASP: Neuronal wiskott Aldrich syndrome; ARP2/3: Actin related proteins 2/3; ROCK: Rho-associated kinase; NSCLC: Non small cell lung carcinoma; DPPIV: Dipeptidyl peptidase-4; SCID: Severe combined immunodeficiency; HSC: Heat shock protein; PCR: Polymerase chain reaction; DMEM: Dulbecco's Modified Eagle Medium; ECIS: Electric cell-substrate impedence sensing; BSS: Buffered saline solution; FITC: Fluorescein isothiocyanate; TRITC: Tetramethyl Rhodamine Isothiocyanate; ECL: Enhanced chemiluminescence; Q-PCR: Quantitative polymerase chain reaction; FAM: 5-carboxyfluorescein; GAPDH: Glyceraldehyde 3-phosphate dehydrogenase; CK-19: Cytokeratin-19; ER: Oestrogen receptor; RT-PCR: Reverse-transcriptase polymerase chain reaction; CEA: Carcino Embryonic Antigen; OPN: Osteopontin; LOX: Lysyl oxidase; EDTA: Ethylenediaminetetraacetic acid; ECM: Extracellular membrane.

## Competing interests

The authors declare that they have no competing interests.

## Authors’ contributions

JJ and WGJ contributed to concept design. JJ established the FAP-α stable cell lines, carried out most of the molecular and functional studies, contributed to data analysis and interpretation, and drafted the manuscript. TAM contributed to concept design, data analysis and interpretation and manuscript writing. LY performed apoptosis assays. TAM, WGJ and LY contributed to preparation of the breast cancer patient samples. All authors read and approved of the final manuscript.
